# Understanding Medicaid Estate Recovery: The Experience of North Carolina and Policy Implications for Future Reforms

**DOI:** 10.1111/1475-6773.70141

**Published:** 2026-06-13

**Authors:** Amanda Spishak‐Thomas, Emma Sandoe, Heather Howard

**Affiliations:** ^1^ Center for State Health Policy Rutgers University New Brunswick New Jersey USA; ^2^ Oregon Health Authority Salem Oregon USA; ^3^ School of Public and International Affairs Princeton University Princeton New Jersey USA

**Keywords:** aging, homeownership, long‐term care, medicaid, racial disparities

## Abstract

**Objective:**

To estimate the prevalence of Medicaid estate recovery in North Carolina.

**Study Setting and Design:**

We descriptively analyzed the number of estates, amount recovered, and hardship waiver applications using North Carolina public records data.

**Data Sources and Analytic Sample:**

The data contained information on the number of estates and amount recovered through Medicaid estate recovery between 2017 and 2021 (*n* = 2975). Additionally, we analyzed hardship waiver application data for residents who applied and had their application processed between 2018 and 2021 (*n* = 301).

**Principal Findings:**

We found approximately $83 billion was recovered from beneficiaries between 2017 and 2021, or 0.6% of the total cost of North Carolina's annual Medicaid program and just 0.9% of the fee‐for‐service annual long‐term services and supports Medicaid budget. We found that Black homeowners were more likely to have lower value estates recovered, and overall, more money was recovered from white estates.

**Conclusion:**

While states hope Medicaid estate recovery may balance Medicaid spending through increased revenue, our findings demonstrate that these efforts may be insufficient. Policymakers should opt for strategies outside of the Medicaid program that can more precisely target high‐net‐worth individuals instead of policies like estate recovery that disproportionately impact low‐income families.

## Callout Box

- What is known on this topic
  ○ Since 1993, Medicaid estate recovery requires states to recoup costs associated with long‐term services and supports from certain deceased Medicaid beneficiaries.
  ○ All 50 states and the District of Columbia have implemented Medicaid estate recovery policies, with some states carrying out more aggressive recovery than others.
  ○ Medicaid is a means‐tested program with strict eligibility requirements, meaning most Medicaid beneficiaries have few assets to begin with and report a net wealth of less than $48,500 nationwide.
- What this study found
  ○ We observed white Medicaid beneficiaries experience higher average amounts recovered from their estates compared to Black beneficiaries.
  ○ The value of homes owned by Black families is substantially less than white homeowners, which may explain in part why more money was recovered from white homeowners.
  ○ Relative to the number of annual recoveries, hardship waiver applications were underutilized, especially considering those submitted had a high rate of being granted for both white and Black applicants.

## 1 Introduction

Medicaid's design as both a state and federal program ensures that states co‐finance the program and are incentivized to constrain spending. The 1993 Medicaid estate recovery policy allows states to seek repayment for the use of Medicaid funds used for long‐term services and supports (LTSS) for adults 55 and older after they die [1, 2, 3]. Medicaid debt becomes a debt of the beneficiary's estate, allowing states to recover costs by seizing a beneficiary's home, one of the few remaining assets that a Medicaid beneficiary can possess while qualifying for benefits [3]. Simultaneously, homeownership is important for older adults as they become more reliant on wealth rather than income for support [4]. Homeownership represents more than stable housing, but also the freedom to make choices about care, with many older adults choosing to remain home as long as possible [5]. It also affects the financial security of adult children, shoring up an inheritance after their parents' death.

Initially designed to ensure that high‐income individuals were not benefiting from taxpayer funds intended for low‐income individuals [6] and those with the resources to pay for long‐term care do so [3], estate recovery may instead contribute to wealth inequities, placing a particular burden on Black homeowners through the pursuit of modest homes [7]. Those with more resources—often white, wealthy families—have access to attorneys who can draft estate planning documents that shield homes and other large assets (e.g., future inheritances) for the next generation, while still appearing to qualify for Medicaid [7, 8, 9]. With estate recovery happening in probate courts, a process which varies by state, research on estate recovery has been limited because of the lack of aggregated, national data [10]. For some, recovery of remaining assets may cause undue strain. In response, states offer families the opportunity to apply for a hardship waiver. In such cases, Medicaid cannot make a claim that requires the home to be sold. The Supplemental Appendix provides more details about North Carolina's hardship process.

Although states were granted flexibility in program design, policy specifications remained mostly stagnant until recently when several states implemented changes, including benefits pursued for recovery, cost‐effectiveness (i.e., the ratio of administrative costs to collections), and hardship practices. For example, in 2023, North Carolina increased the estate value threshold from $5000 to $50,000 [6, 11]. Two known prior studies have investigated the implementation of recovery policies, but neither identify who is targeted by estate recovery [7, 10]. To our knowledge, this is the first case study of Medicaid estate recovery using public records data that include information on race, zip code, number and amounts recovered, and number and determination of hardship waiver applications, filling an important literature gap about who estate recovery may be affecting the most.

Given existing wealth inequities nationwide where white homeowners have almost double the median home equity as Black and Hispanic homeowners [12] and considering nearly 40% of Medicaid enrollees in North Carolina are Black, compared to 55% white [13], we hypothesize that estate recovery may disproportionately impact Black families. This study uses novel data to descriptively answer three questions. First, what is the number of estates recovered, and the amount recovered from those estates? Secondly, how many hardship applications were submitted and what were the determinations? Lastly, among those affected, are there any characteristics that might predispose someone to the policy? Racial differences in hardship waiver acceptances could be suggestive of bias during the application review process, which would be informative to state administrators. Findings from this study can inform policymakers implementing changes to the 30‐year‐old policy to improve cost‐effectiveness and equity.

## 2 Methods

### 2.1 Data Sources

The primary data for this analysis were obtained from the North Carolina Department of Health and Human Services (DHHS), available from a public records request to state administrators. State variation in estate recovery extends beyond policy specifics and includes variation in data collected, where states choose which sociodemographic data, if any, to track alongside amount and frequency of recovered estates. This analysis leverages an existing relationship with North Carolina DHHS and a year‐long data acquisition process to take advantage of a state with more robust demographic data linked to estate recoveries and hardship waiver applications.

### 2.2 Study Population and Measures

The data contained information on the number and amount recovered between January 1, 2017 and December 31, 2021, per 3‐digit zip code. To assess the prevalence of fully recovered estates, the numerator was estates subject to recovery over a five‐year period (*n* = 2975), and the denominator was deceased Medicaid beneficiaries. Additionally, we used zip code‐level data on the number and determination of hardship waiver applications for residents who applied and had their application processed between January 1, 2018 and Dec 31, 2021 (*n* = 301). Lastly, we merged relevant zip code characteristics from the US Census Bureau to understand the relationship between Medicaid estate recovery and regional demographic and geographic characteristics. Appendix A1 in the Supporting Information provides details about how the dataset was structured.

The dependent variables were the number and amount (e.g., value) of recovered estates by North Carolina's estate recovery policy in US dollars as well as the number and determination of hardship waiver applications (granted, denied, deferred). Given the limitations of the data, we calculated the average recovered per person using the total amount recovered per year and 3‐digit zip code divided by the total number of estates recovered in that year. North Carolina's DHHS provided a variable for race that was self‐reported at application, thus differs from typical US Census categories (white, Black, Asian, American Indian, Black/Indian, Black/white, and unknown). Due to small population sizes, we recoded race as a 3‐level categorical variable (white, Black, and other). Similarly, our partnership with DHHS enabled access to the first 3‐digits of zip codes, which we used alongside definitions provided by the Federal Office of Management and Budget to create a 3‐level urbanicity variable that defined zip codes as predominantly urban, rural, or micropolitan.

### 2.3 Statistical Analysis

We conducted a descriptive analysis to identify trends over time indicating the annual prevalence of estate recovery. The total and average amount recovered from estates were documented by year overall and stratified by race. We identified the number of hardship waiver applications and determinations of applications by year overall and stratified by race. Then, we analyzed the frequency of estate recovery, amount (total and average) recovered from estates, and prevalence of hardship waiver determinations (granted, denied, and deferred), overall and by race. Wald tests were used to identify significant differences by race. Finally, we used linear regression models to assess the association of sociodemographic (race) and geographic characteristics (urbanicity, median home value, neighborhood educational attainment, neighborhood Medicaid insurance) with the average amount recovered. The neighborhood‐level variables represent the percent of the population with a high school diploma or equivalent and the percent enrolled in Medicaid. For hardship waivers, we ran logistic regression models with the same set of variables to understand which characteristics may be associated with having a hardship application granted (or denied).

## 3 Results

**FIGURE 1:**
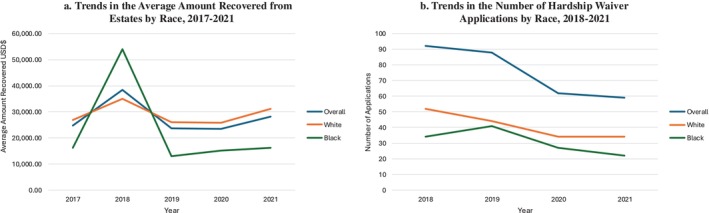
(a) Trends in the average amount recovered from estates by race, 2017–2021. Analysis of 2017–2021 North Carolina Public Records data of the average amount from recovered estates in North Carolina. The average amount recovered variable represents the average amount recovered per person for all years (the total amount recovered from all estates divided by the total number of estates recovered). Numbers presented are unweighted. (b) Trends in the number of hardship waiver applications by race, 2018–2021. Analysis of 2018–2021 North Carolina Public Records data of the number of hardship waiver applications submitted by surviving relatives of Medicaid beneficiaries. Numbers presented are unweighted totals.

Overall, average amounts recovered ranged from a low of $23,372.42 in 2020 to a high of $38,424.94 in 2018 (Figure 1a). There were higher average amounts recovered from the estates of white beneficiaries compared to Black beneficiaries. For white beneficiaries, the average amounts were similar over time ranging from a 2018 high of $35,037.70 to 2019 and 2020 lows of just under $26,000. The highest average amount recovered from Black beneficiaries was in 2018 with almost $55,000 collected, with an annual low of less than $15,000 per person in 2019. The 2018 peak may be driven by an outlier where a single Black beneficiary had $757,822.99 recovered that year. Appendix A3 in the Supporting Information displays trends in the median average amount recovered from estates by race. Overall trends are similar to Figure 1a. For white beneficiaries, the median amounts range from a 2018 high of $34,589.52 to a low in 2020 of $19,878.84. Black beneficiaries also have a median low in 2020 of $9928.42 and a high in 2018 of $44,147.24. There were 301 hardship waiver applications processed during the study period: 164 by white applicants and 124 from Black applicants (Figure 1b). Overall, a total of $83,183,922.91 was collected from 2975 North Carolina estates between 2017 and 2021 (Table 1). An average of $27,960.98 was collected per person between 2017 and 2021. There were statistically significant differences in the number and value of recovered estates between white and Black beneficiaries. Nearly 8 in 10 estates were recovered from white beneficiaries, compared to just under 20% of estates recovered from Black beneficiaries (*p* < 0.001). Approximately 84% ($69,754,514.03) was collected from white estates. An average of $29,173.78 was collected from white Medicaid beneficiaries compared to $22,813.60 from Black Medicaid beneficiaries (*p* < 0.001).

**TABLE 1:** Estate recoveries and hardship waivers overall and by race.

			Race			
	Total		White		Black	
Estate recoveries, 2017–2021						
Number of estates recovered, no. %	2975		2391	80.37%***	581	19.53%
Total recovered, $ (SD)	83,183,922.91 (549,069)		69,754,514.03 (638,552)		13,254,704.25 (179,157)	
Total recovered, $, median	197,131.93		525,291.20		95,690.31	
Average recovered, $, mean (CI)	27,960.98		29,173.78***		22,813.60	
	(27,297.99 to 28,623.97)		(28,822.68 to 29,524.88)		(19,801.39 to 25,825.82)	
Average recovered, $, median	26,503.61		27,831.38		16,649.93	
Hardship waivers, 2018–2021						
Total applications, no., %	301		164	54.49%	124	41.20%
Granted, no., %	136	45.18%	69	42.07%	60	48.39%
Denied, no., %	103	34.22%	58	35.47%	42	33.87%
Deferred, no., %	62	20.60%	37	22.56%	22	17.74%

*Note:* Analysis of North Carolina Public Records data containing the number and value of estates recovered (2017–2021) and the number and outcome of hardship waiver applications (2018–2021). The average amount recovered variable represents the average amount recovered per person for all years (the total amount recovered from all estates divided by the total number of estates recovered). Numbers presented are unweighted. Percentages represent the percent as it relates to overall number. Any cell sizes with fewer than 10 observations were omitted from analysis. Wald tests were conducted to assess the *p*‐value (i.e., the difference in percentages or average amounts). For hardship waivers, there were no significant differences observed between groups. 95% confidence intervals in parentheses ***p < 0.001.

Of the 301 total hardship waiver applications submitted (Exhibit 2), 136 were granted (45.2%) and 103 were denied (34.2%). Over half of the applications (164; 54.6%) were submitted by white applicants compared to 124 (41.2%) Black applicants. Nearly half of white applicants were granted a hardship waiver (42.1%) and 35.5% were denied. Black applicants had a slightly higher likelihood of having their applications granted (48.4%), but differences were not statistically significant.

Hardship application trends over time are shown in Appendix A4 in the Supporting Information. Between 2018 and 2021, there was a sharp decline in granted applications and an overall increase in the deferral of hardship waiver applications from 0 in 2018 to 28 deferrals, or 47.5% of total applications in 2021. The number of denied hardship waivers declined steeply between 2019 and 2020 but rebounded in 2021.

In the linear regression models, the average amount recovered from the estate of a Black beneficiary was $5023 less than the amount recovered from a white beneficiary (95% CI: −6676.04 to −3370.33; *p* < 0.01) (Table 2). Considering the composition of the population at the zip code level, a one percentage point higher share of Black residents in a zip code was associated with a significantly lower average amount recovered per person by $19,341 (95% CI: −28,337.29 to −10,344.33; *p* < 0.01). Further, a one percentage point higher share of the zip code population enrolled in Medicaid was associated with a $37,746 higher average amount recovered from estates (95% CI: −5139.24 to 80,631.19; *p* < 0.1). A one percentage point higher share of the zip code population with at least high school educational attainment was associated with a lower average estate recovery amount by $67,846 per person in adjusted analyses (95% CI: −105,489.90 to −30,201.94; *p* < 0.01).

**TABLE 2:** Individual and geographic characteristics associated with the average amount recovered from individual estates, 2017–2021.

	Average amount recovered from estates
Characteristics	($)
White	[Reference]
Black	−5023.00***
	(−6676.04 to −3370.33)
Zip code level characteristics	
Predominantly urban	[Reference]
Predominantly rural	2065.00
	(−1641.84 to 5772.66)
Predominantly micropolitan	−1841.00
	(−5220.50 to 1537.78)
Average median home value	0.02
	(−0.01 to 0.05)
% High school graduate	−67,846.00***
	(−105,489.90 to −30,201.94)
% Medicaid	37,746.00*
	(−5139.24 to 80,631.19)
% Black population	−19,341.00***
	(−28,337.29 to −10,344.33)
Observations	2975

*Note:* Authors' analysis of 2017–2021 North Carolina Public Records data. Linear regression results presented. The average amount recovered variable represents the average amount recovered per person for all years (the total amount recovered from all estates divided by the total number of estates recovered). The number of observations represents 2975 unique recovered estates. 95% confidence intervals in parentheses. *p < 0.1, **p < 0.05, ***p < 0.01.

In our logistic regression models, there were no statistically significant relationships between granted or denied hardship waivers and population characteristics (Appendix A2 in the Supporting Information).

## 4 Discussion

In this study of North Carolina efforts to recover monies from estates used by Medicaid beneficiaries for long‐term services and supports (LTSS), we found that estate recovery recouped less than 1% of the state's Medicaid budget. Of the estates recovered, estates of Black homeowners were of lower value than their white counterparts, therefore the highest amounts recovered were from the estates of white homeowners. Neighborhoods with high concentrations of Medicaid enrollment (e.g., low‐income neighborhoods) were associated with higher estate recovery. Relative to the number of annual recoveries, hardship waiver applications were underutilized, especially considering those that were submitted, had a high rate of being granted for both white and Black applicants.

On average, the value of homes owned by Black families (average equity of $27,000) was less than white homeowners (average equity of $73,000) in the US [14]. The difference in valuation may explain why significantly more money was recovered from white homeowners in North Carolina. States may have more to gain from white homeowners whose homes tend to be worth more, on average, than Black homeowners. On average, just $22,000 was recovered from Black Medicaid beneficiaries, compared to almost $30,000 per white beneficiary. These averages do not consider differences in wealth accumulation between white and Black Medicaid beneficiaries. For some Black families, $22,000 may be a more meaningful amount than for white families given persistent disparities in wealth.

While North Carolina offers insight into estate recovery across the country, it is likely more generalizable to states with similar characteristics. We might expect similar results in a comparable regional state such as Georgia, which has a similar composition of rural and Black residents and has made recent changes to their policy, increasing the cost‐effectiveness threshold to not pursue estates valued below $25,000 (North Carolina uses $50,000 for their home value minimum) [15]. There is some evidence that Georgia processes an equivalent volume of hardship waivers with approximate rates of granted applications [7]. South Carolina offers another example where findings may generalize based on analogous populations living below the federal poverty level as well as rates of uninsurance and median home values [16]. Relatedly, Missouri, Oregon, South Carolina, Michigan, Virginia, and Wisconsin have proportional fee‐for‐service LTSS spending, but only Missouri recovers an equivalent amount [7]. Overall, recovery trends may vary in areas that are dissimilar based on homeownership rates, urbanicity, and Medicaid enrollment as well as variation in recovery policies like cost‐effectiveness thresholds and home value minimums. More research using public records data from other states would clarify the scope of estate recovery and hardship waivers.

This study has several other limitations. The demographic data available were limited, and did not include age, ethnicity (e.g., Hispanic or Latino), and gender of those who experienced estate recovery, which would strengthen the analysis. Our analysis relied on data that did not capture individual‐level variation and was limited to only those who had their estates recovered during the study period. We did not have data for processes that may still be in probate court and would not otherwise be observable, which may lead to right‐censoring of the data (i.e., processes that have not yet concluded, thus cannot be observed). While we approximated the average amount recovered per person, variation may exist in the actual amount recovered per estate, skewing results in either direction. Because the data were at the zip code level, findings were limited to descriptive observations of trends and associations, and we cannot make causal conclusions.

Our study provides a starting point to approximate the scope of Medicaid estate recovery based on what is observable using novel data. Medicaid costs the state of North Carolina an average of $3.5 billion annually, with approximately $2.4 billion spent on fee‐for‐service LTSS [7, 17]. We find that the policy recovered nearly $83,200,000 from Medicaid beneficiaries between 2017 and 2021, or 0.59% of the total cost of the state's annual Medicaid program and just 0.85% of the fee‐for‐service annual LTSS Medicaid budget. While states hope estate recovery may balance Medicaid spending through increased revenue, our findings demonstrate that these efforts may be insufficient. Medicaid is a means‐tested program with strict eligibility, resulting in a majority of Medicaid beneficiaries with few assets reporting a net wealth of less than $48,500 nationwide [7]. Policymakers should opt for strategies outside of the Medicaid program that can more precisely target high‐net‐worth individuals through mechanisms like tax increases on high income earners and large inheritances instead of policies like estate recovery that disproportionately impact low‐income families. This North Carolina case study offers unique insight into one state's experience of Medicaid estate recovery, but more research is needed to understand the policy in other states.

## Funding

The authors have nothing to report.

## Conflicts of Interest

The authors declare no conflicts of interest.

## Supporting information


**Appendix A1.** Supplementary appendix.
**Appendix A2**. Individual and geographic characteristics associated with hardship waiver determinations, 2018–2021.
**Appendix A3**. Trends in the median average amount recovered from estates by race, 2017–2021.
**Appendix A4**. Hardship waiver determinations, 2018–2021.

## Data Availability

The data that support the findings of this study are available from North Carolina Department of Health and Human Services. Restrictions apply to the availability of these data, which were used under license for this study. Data are available from the author(s) with the permission of North Carolina Department of Health and Human Services.
