# Supplementary material for: Understanding Medicaid Estate Recovery: The Experience of North Carolina and Policy Implications for Future Reforms
Source: Health Serv Res. 2026 Jun 13;61(3):e70141. doi: 10.1111/1475-6773.70141 (PMC13263700; doi:10.1111/1475-6773.70141)
Supplement: Supplementary file 1 — Appendix A1. Supplementary appendix. Appendix A2. Individual and geographic characteristics associated with hardship waiver determinations, 2018–2021. Appendix A3. Trends in the median average amount recovered from estates by race, 2017–2021. Appendix A4. Hardship waiver determinations, 2018–2021. [file HESR-61-0-s001.docx]

Supplemental Appendix

Appendix A1.

***Hardship Waivers***

Every state is required to acknowledge situations where estate recovery may cause undue hardship. The definition and criteria for what constitutes undue hardship varies by state.^1^ North Carolina’s hardship waiver policy allows children, grandchildren, siblings, and the children of siblings to apply for a waiver if recovery causes “undue hardship,” defined in three ways: 1) if the property is the sole source of income and the gross income is below 200% of the federal poverty level, 2) if recovery would result in the sale of property included in the estate of the decedent and the applicant resides in the property, 3) if recovery results in the sale of property included in the estate of the decedent and the applicant shares a tenancy in the common interest of the property.^7^ In other words, if the estate includes an income-producing asset like an Airbnb or working ranch or the assets have been protected by a trust, the house immediately belongs to an heir and will not pass through probate court. Protecting assets in a trust is a strategy used by families with access to estate planning resources to evade policies like recovery. However, not every family has the privilege or resources to plan ahead, which may lead to disparities in inherited assets upon the death of a Medicaid beneficiary.^5^

In North Carolina, surviving relatives are notified about Medicaid estate recovery within 30 days of the death of the Medicaid beneficiary. This process is followed by the state determining the amount owed to Medicaid, at which point the surviving relatives will be re-notified of Medicaid’s claim on the estate and can apply for a hardship waiver. The next of kin can apply for a waiver within 60 days of the date of notice of the Medicaid claim if estate recovery procedures cause “undue hardship” on surviving relatives. Within 90 days of a hardship waiver application, the applicant will be notified of the determination and in the case of a denied application, it can be appealed within another 60 days. The creation of hardship waivers signals an understanding of the strain estate recovery can cause a family. Hardship waivers offer a way out for families who would otherwise lose assets to estate recovery in the wake of the death of a loved one. However, just as with estate recovery itself, it is unclear how many families apply for hardship waivers annually and little knowledge of the demographic and geographic characteristics of applicants.

A deferral, also sometimes referred to as time-limited undue hardship waivers, is when the state approves a partial waiver. The partial waiver only applies to some assets in the estate when the total assets of the estate are less than $5,000 or the total Medicaid payments eligible for recovery are less than $3,000 – below the cost effectiveness threshold. In this event, the state defers the claim against the estate until the conditions above are no longer met, at which point they may pursue recovery.

***Data Structure***

*Estate Recoveries*

The data contain three variables: 3-digit zip code, year, and race. Each combination of the three variables included the number of estates and the total value recovered for the people in that group (n=195 groupings of 3-digit zip codes by race and year). One row of data may have zip code 270, year 2021, race Black, 5 estates recovered, and a total of $100,000 recovered (from those 5 estates). Another row of data may have the same zip code, 270, the same year 2021, but with White individuals who had 31 estates recovered, and $1,000,000 recovered (from those 31 estates). Analyses were restricted to residents of North Carolina for the duration of the study period: those who moved out of state were dropped from the analytic sample. The analytic sample consists of residents whose estates were recovered during the study period and who were residents for the duration of the period, representing 2,975 total number of estates.

Visual example of estate recovery data structure

| 3-digit zip code | Year | Race | # of Estates Recovered | Amount Recovered |
| --- | --- | --- | --- | --- |
| 270- | 202 | Black | 5 | $100,000 |
| 270- | 2021 | White | 31 | $1,000,000 |

*Hardship Waivers*

The data had a similar structure to the number and amount data, with groupings by 3-digit zip code, race, and year. Unlike the data containing estate recoveries, each row of the hardship waiver data represented the determination of one hardship waiver application submitted by a North Carolina resident during the study period (n=301 applications). One row of data may have zip code 275, year 2020, race White, and a granted hardship waiver application. The next row may have the same zip code 275, year 2020, race Black, and a denied hardship waiver application.

Visual example of hardship waiver data structure

| 3-digit zip code | Year | Race | Determination |
| --- | --- | --- | --- |
| 275- | 2020 | White | Granted |
| 275- | 2020 | Black | Denied |

*U.S. Census Bureau*

We linked U.S. Census Bureau data at the ZIP Code Tabulation Area (ZCTAs) level to the public records data using the 3-digit zip codes. The data were used to examine the relationship between zip code characteristics (e.g., geographic characteristics) and outcomes to determine the geographic differences that might make someone more or less likely to be affected by estate recovery. These geographic characteristics were treated as independent variables and included a 3-level categorical urbanicity variable (urban, micropolitan, or rural), percent of the population enrolled in Medicaid, percent of the population that identify as non-Hispanic Black, percent with a high school diploma or equivalent, and average median home value. Given 3-digit zip codes are broad regions that encompass many 5-digit zip codes, averages were calculated across comprising zip codes for each geographic characteristic. The data are structured such that each 3-digit zip code is assigned a rate of geographic characteristic or value measure. Thus, if a respondent lives in a zip code that starts with 270, they are assigned the characteristics from 270. This is a critical layer given the large rural population and significant urban-rural disparities in health and wealth in North Carolina.

**Appendix A2. Individual and Geographic Characteristics Associated with Hardship Waiver Determinations, 2018-2021**

|  | Granted Hardship Waiver | Denied Hardship Waiver |
| --- | --- | --- |
| Characteristics | (OR) | (OR) |
| White | 1 [Reference] | 1 [Reference] |
| Black | 1.38 | 0.90 |
|  | (0.78 to 2.43) | (0.52 to 1.56) |
| Other | 1.77 | 0.51 |
|  | (0.47 to 6.72) | (0.13 to 2.10) |
| Zip Code Level Characteristics |  |  |
| Predominantly urban | 1 [Reference] | 1 [Reference] |
| Predominantly rural | 0.66 | 0.64 |
|  | (0.15 to 2.94) | (0.15 to 2.69) |
| Predominantly micropolitan | 0.38 | 0.95 |
|  | (0.09 to 1.58) | (0.25 to 3.66) |
| Average median home value | 1.00 | 1.00 |
|  | (1.00 to 1.00) | (1.00 to 1.00) |
| % High school graduate | 0.00 | 4984.05 |
|  | (0.00 to 484.66) | (0.00 to 1.09e+10) |
| % Medicaid | 0.34 | 0.00 |
|  | (0.00 to 4434630.00) | (0.00 to 438.29) |
| % Black population | 0.14 | 8.99 |
|  | (0.00 to 5.90) | (0.23 to 355.42) |
| Observations | 301 | 301 |
| 95% confidence intervals in parentheses |  |  |

Notes: Authors’ analysis of 2017-2021 North Carolina Public Records data. Logistic regression results presented. The number of observations represents 301 unique applications.

**Appendix A3. Trends in the Median Average Amount Recovered from Estates by Race, 2017-2021**


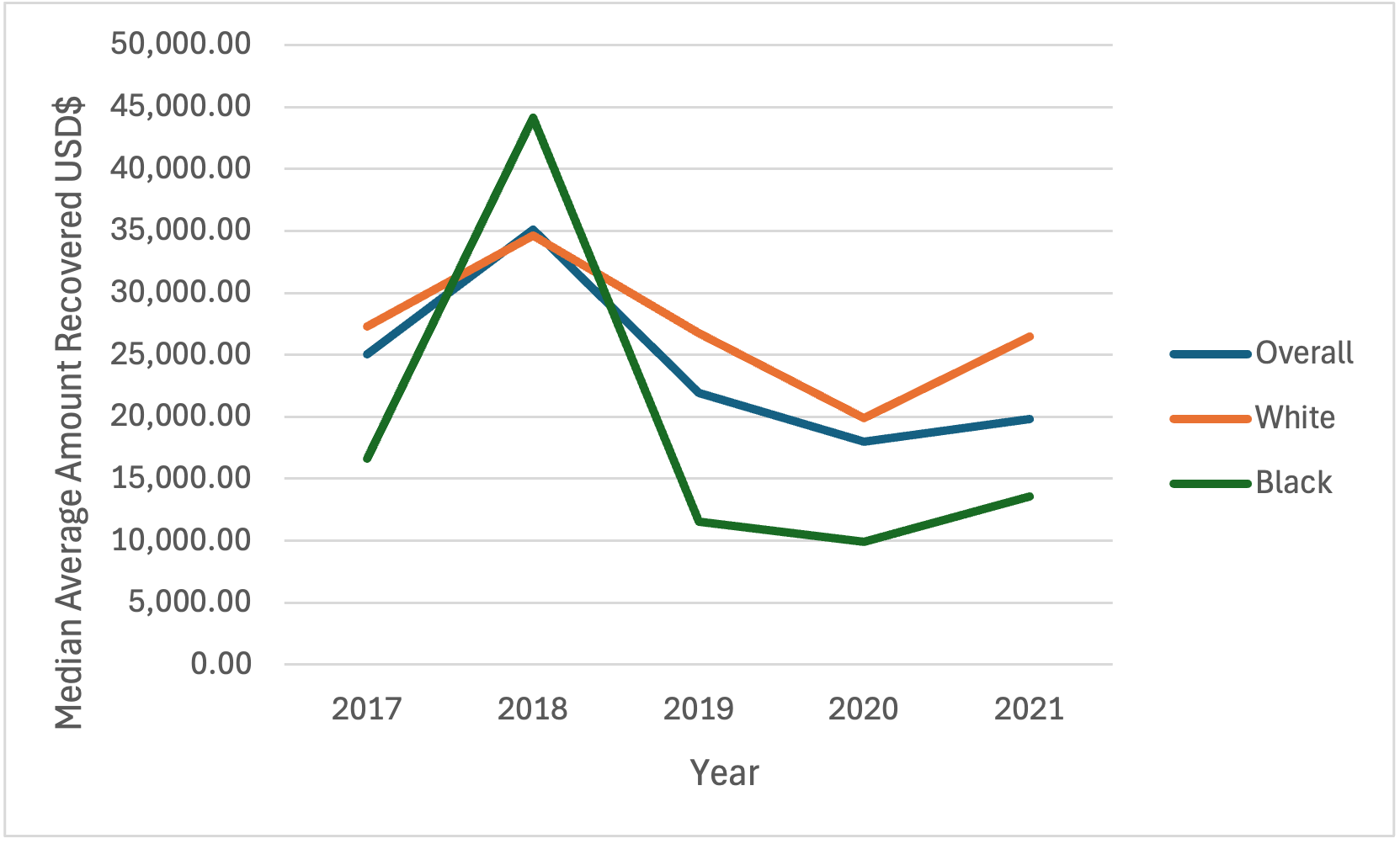


Notes: Analysis of 2017-2021 North Carolina Public Records data displaying the median average amount from recovered from estates in North Carolina. The average amount recovered variable represents the average amount recovered per person for all years (the total amount recovered from all estates divided by the total number of estates recovered). Numbers presented are unweighted medians.

**Appendix A4. Hardship Waiver Determinations, 2018-2021**


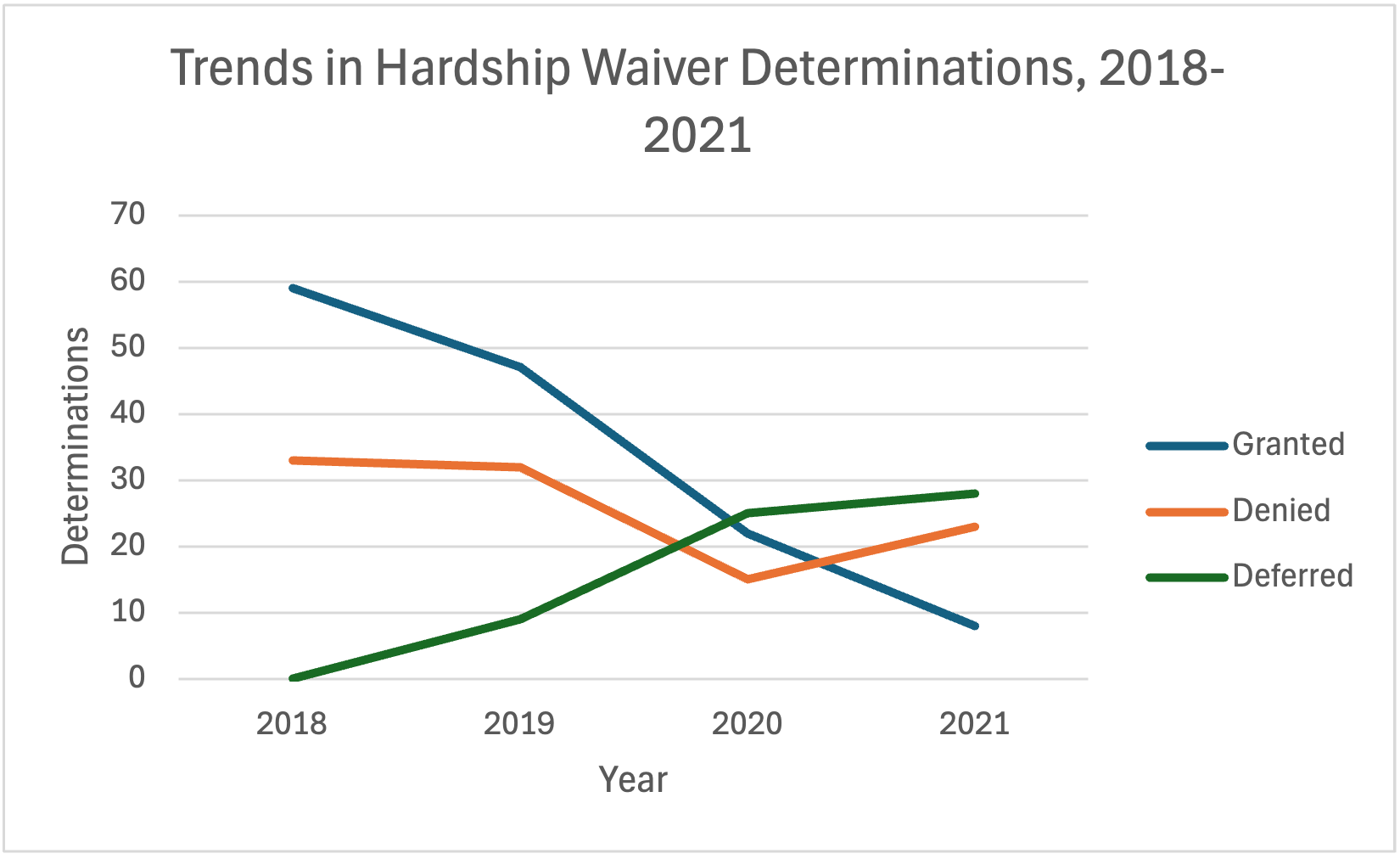


Notes: Analysis of 2018-2021 North Carolina Public Records data of the determination of hardship waiver applications submitted by surviving relatives of Medicaid beneficiaries in North Carolina. Numbers presented are unweighted totals.
